# A DNA methylation-based test for esophageal cancer detection

**DOI:** 10.1186/s40364-020-00248-7

**Published:** 2020-11-25

**Authors:** Sofia Salta, Catarina Macedo-Silva, Vera Miranda-Gonçalves, Nair Lopes, Davide Gigliano, Rita Guimarães, Mónica Farinha, Olga Sousa, Rui Henrique, Carmen Jerónimo

**Affiliations:** 1 Cancer Biology & Epigenetics Group – Research Center, Portuguese Oncology Institute of Porto, Rua Dr António Bernardino de Almeida, 4200-072 Porto, Portugal; 2 Department of Pathology, Portuguese Oncology Institute of Porto, Rua Dr. António Bernardino de Almeida, Porto, 4200-072 Portugal; 3 Department of Radiation Oncology, Portuguese Oncology Institute of Porto, Rua Dr. António Bernardino de Almeida, Porto, 4200-072 Portugal; 4 Department of Pathology and Molecular Immunology, Institute of Biomedical Sciences Abel Salazar– University of Porto , Rua de Jorge Viterbo Ferreira, 228, Porto, 4050-313 Portugal

**Keywords:** Esophageal Cancer, DNA methylation, Early detection; treatment response

## Abstract

**Background:**

Esophageal cancer (ECa) is the 7th most incident cancer and the 6th leading cause of cancer-related death. Most patients are diagnosed with locally advanced or metastatic disease, enduring poor survival. Biomarkers enabling early cancer detection may improve patient management, treatment effectiveness, and survival, are urgently needed. In this context, epigenetic-based biomarkers such as DNA methylation are potential candidates.

**Methods:**

Herein, we sought to identify and validate DNA methylation-based biomarkers for early detection and prediction of response to therapy in ECa patients. Promoter methylation levels were assessed in a series of treatment-naïve ECa, post-neoadjuvant treatment ECa, and normal esophagus tissues, using quantitative methylation-specific PCR for *COL14A1*, *GPX3,* and *ZNF569*.

**Results:**

*ZNF569* methylation (*ZNF569me)* levels significantly differed between ECa and normal samples (*p* < 0.001). Moreover, *COL14A1* methylation (*COL14A1me)* and *GPX3* methylation (*GPX3me)* levels discriminated adenocarcinomas and squamous cell carcinomas, respectively, from normal samples (*p* = 0.002 and *p* = 0.009, respectively). *COL14A1me* & *ZNF569me* accurately identified adenocarcinomas (82.29%) whereas *GPX3me & ZNF569me* identified squamous cell carcinomas with 81.73% accuracy. Furthermore, *ZNF569me* and *GPX3me* levels significantly differed between normal and pre-treated ECa.

**Conclusion:**

The biomarker potential of a specific panel of methylated genes for ECa was confirmed. These might prove useful for early detection and might allow for the identification of minimal residual disease after adjuvant therapy.

## Background

Esophageal cancer (ECa) is the 7th most common malignancy and the 6th cause of cancer-related mortality worldwide [1]. ECa comprises two main histological subtypes: adenocarcinoma and squamous cell carcinoma, the most prevalent type [2]. Although therapeutic improvements have increased ECa survival rates [3], curative-intent treatment options remain limited to surgery, chemotherapy (ChT), and radiotherapy (RT), either alone or in trimodal therapy [4]. Furthermore, most tumors are diagnosed at an advanced stage, entailing very low 5-year survival rates, ranging from 5 to 20% [5, 6]. Intensive research was, thus, carried out to find alternative treatment strategies to prolong life expectancy and quality of life (QoL) for ECa patients. Currently, neoadjuvant treatment with ChT and/or RT is the standard of care for patients with locally advanced disease [2]. Nevertheless, surgery is often associated with decreased QoL due to complications and comorbidities [7, 8].

Epigenetic alterations, including their key players, have emerged as promising biomarkers for ECa. Among those, gene methylation is the most extensively studied epigenetic modification. Although esophageal adenocarcinomas (EA) and esophageal squamous cell carcinomas (ESCC) display different morphological and molecular features, several studies have shown that methylation is an early event in both histotypes, already present in premalignant lesions [9–12]. Thus, promoter methylation of specific genes might be used to discriminate normal from cancerous esophageal cells, enabling the detection of disease at early stages, increasing the likelihood of curative treatment. Except for *CDKN2A* and *APC*, which are commonly aberrant methylated genes in both EA and ESCC, a specific methylome has been reported for each histotype [13]. The importance of these alterations is further highlighted by the numerous reports correlating aberrant DNA methylation with ECa patient prognosis [14, 15].

## Methods

Based on the literature evidence, we aimed to identify and validate methylome alterations that might constitute biomarkers for early detection, as well as identification of minimal residual disease after neoadjuvant treatment, eventually precluding the need for esophagectomy. Hence, the studies selected from the literature clearly reported sensitivity and specificity for the detection of ECa or Barrett’s Esophagus using DNA methylation-based biomarkers (Additional Table 1). Studies with a small cohort of patients [*n* ≤ 40 for each group (cases and controls)] were also excluded. Furthermore, only genes with a specificity higher than 98% were selected for testing. Of the remaining 5 genes, no specific primers were obtained to test *ZNF345* and *EPB41L3*. Hence, promoter methylation levels of three genes selected [16, 17] - *GPX3* (*glutathione peroxidase 3*), *COL14A1* (*collagen type XIV alpha 1 chain*), and *ZNF569* (*zinc finger protein 569*) - were tested in a series of ECa and normal esophageal tissues.

### Patients and samples collection

A total of 124 formalin-fixed paraffin-embedded (FFPE) tissues samples from patients diagnosed with ECa between 2007 and 2017 at the Portuguese Oncology Institute of Porto (IPO-Porto) were included in this study (Table 1). Among the tumor samples, 88 were collected before any treatment previous surgery, and 36 were collected after neoadjuvant treatment (ChT and/or RT). Additionally, 56 FFPE tissues samples of normal esophagus from patients diagnosed with gastric carcinoma and without evidence of esophageal cancer were used as control. All samples were archived at the Department of Pathology of IPO-Porto. All the cases were revised by an experienced pathologist and classified according to the World Health Organization (WHO) classification of Tumors of the Digestive System (4th edition) and staged according to the 7th edition American Joint Committee on Cancer (AJCC) system [18, 19]. Relevant clinical data were collected from medical charts. For DNA extraction, a 4 μm section was cut from a representative tissue block and stained with hematoxylin-eosin. Tumor areas were delimited, enabling macrodissection in eight consecutive 8 μm sections. This study was approved by the institutional ethics committee of IPO Porto (CES 202/017).

**Table 1:** Clinicopathological data of Normal Esophagus and Esophageal Tumor patient’s

Clinicopathological Features	Esophageal Tumor Samples		Normal Esophagus
	Naïve Tumors	Post ChT/RT^**a**^ Treatment Tumors	
Patients (no.)	88	36	56
Age median (range)	63 (37–83)	60 (44–73)	66 (36–84)
Sex (no.)			
Man	74	31	37
Woman	14	5	19
Histological subtype (no.)			n.a.
Adenocarcinoma	40	16	
Squamous Cell Carcinoma	48	20	
Localization (no.)			n.a.
Upper	3	–	
Middle	27	5	
Lower	30	18	
GEJ^b^	28	13	
pT Stage/ypT Stage			n.a.
pT1/ypT1	16	5	
pT2/ypT2	14	7	
pT3/ypT3	56	23	
pT4/ypT4	2	1	
pN Stage/ ypN Stage			n.a.
pN0/ypN0	39	17	
pN1/ypN1	17	7	
pN2/ypN2	21	6	
pN3/ypN3	11	6	
Stage			n.a.
I	13	4	
II	32	6	
III	28	19	
IV	15	7	

^a^*ChT/RT* Chemotherapy and/or Radiotherapy, ^b^ *GEJ* Gastroesophageal Junction, *n.a* non- applicable

### Promoter methylation evaluation

DNA extraction from FFPE sections was performed using FFPE RNA/DNA Purification Plus Kit (Norgen Biotek, Thorold, Canada) following the manufacturer’s instructions. DNA concentrations and purity ratios were determined using the NanoDrop Lite spectrophotometer (NanoDrop Technologies, Wilmington, DE, USA) and modified with sodium bisulfite, using the EZ DNA Methylation-Gold™ Kit (Zymo Research, Orange, CA, USA) according to manufacturer’s instructions. For quantitative methylation-specific PCR (QMSP), modified DNA was used as template. Primers to specifically amplify methylated bisulfite converted complementary sequences were used and are listed in Additional Table 2. QMSP reactions were carried out in LightCyler 480 II (Roche, Germany) using 2 μL of modified DNA and 5 μL Xpert Fast SYBR (2X) (GRiSP, Porto, Portugal). All samples were run in triplicate and melting curves were obtained for each case by gene. β-*actin* (*ACTβ*) was used to normalize for DNA input in each sample [20]. To ascertain PCR efficiency, and samples’ quantification, modified CpGenome™ Universal Methylated DNA (Merck Millipore, France) was used in each plate to generate a standard curve. The relative methylation for each gene was calculated by the ratio of mean quantity for the target gene and the mean quantity of *ACTβ*, multiplied by 1000 for easier tabulation.

### Statistical analysis

All the comparisons were performed using non-parametric tests. Specifically, the Kruskal-Wallis test was used for comparisons among three or more groups, whereas the Mann-Whitney U test was used in comparisons between two groups.

To assess biomarker performance, Receiver Operator Characteristic (ROC) curves were constructed for each gene and the Area Under the Curve (AUC) was calculated. The highest value obtained by the ROC curve analysis [sensitivity + (1-specificity)] was established as cut-off to categorize samples as methylated or unmethylated, according to Youden’s J index method [21, 22]. Furthermore, specificity, sensitivity, accuracy, positive likelihood ratio (+LH), and negative likelihood ratio (−LH) were determined. For combination of biomarkers, cases were considered positive if at least one of the individual biomarkers was positive.

Spearman non-parametric correlation was used to assed the correlation methylation levels and age. Disease-specific and disease-free survival curves (Kaplan-Meier with log-rank test) were built for standard clinicopathological variables and categorized methylation status. Disease-specific survival curves and disease-free survival curves (Kaplan–Meier with log-rank test) were computed for standard clinicopathological variables and categorized methylation status.

Two-tailed *P*-values were derived from statistical tests, using a computer-assisted program (SPSS Version 25.0, Chicago, IL), and results were considered statistically significant at *p* < 0.05, with Bonferroni’s correction for multiple tests, when applicable (* *p* < 0.05; ** *p* < 0.01; *** *p* < 0.001; **** *p* < 0.0001; ns – non significant). Graphics were assembled using GraphPad 6 Prism (GraphPad Software, USA).

## Results

### Clinical and pathological data

The most relevant clinical and pathological data are depicted in Table 1. In addition to normal esophageal tissues, ECa cases were segregated into treatment-naïve (samples collected before any treatment) and post-neoadjuvant treatment groups. No significant differences were disclosed concerning age among the three groups of samples (*p* = 0.06).

### Gene promoter methylation levels in naïve ECa tumors vs. normal esophagus samples

**Fig. 1:**
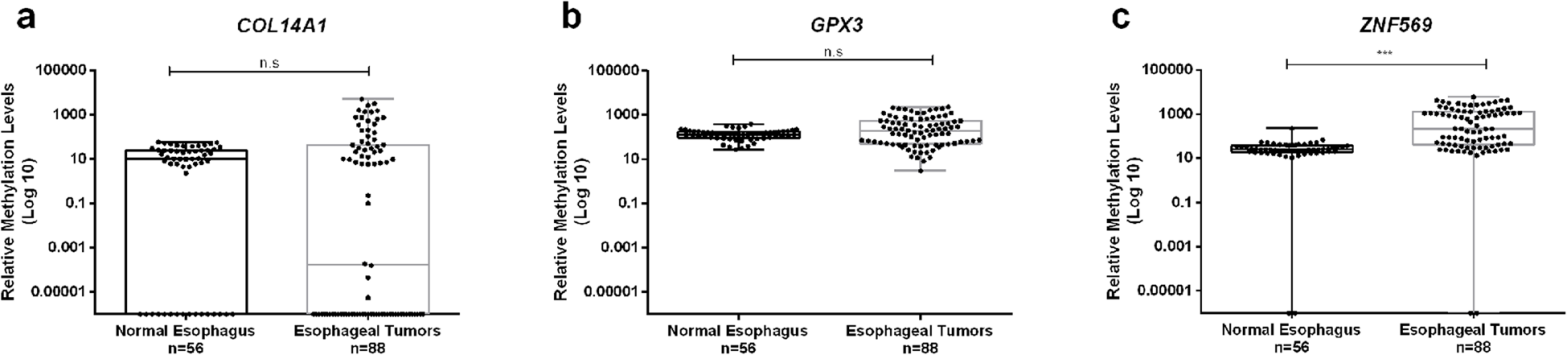
Boxplots with all points of *COL14A1* (**a**), *GPX3* (**b**) and *ZNF569* (**c**) relative methylation levels in the normal esophagus (*n* = 56) and esophageal tumor tissues (*n* = 88). *** *p* < 0.001; n.s- non-significant

To assess performance for ECa detection, *COL14A1* methylation (*COL14A1me), GPX3* methylation (*GPX3me),* and *ZNF569* methylation (*ZNF569me*) levels in naïve tumors (*n* = 88) were compared to normal esophagus samples (*n* = 56). *ZNF569*me levels significantly differed between cancerous and normal samples (*p* < 0.001, Fig. 1c), whereas no significant differences were found for *COL14A1*me and *GPX3*me levels (*p* = 0.382 and *p* = 0.094, respectively, Fig. 1a, b).

**Fig. 2:**
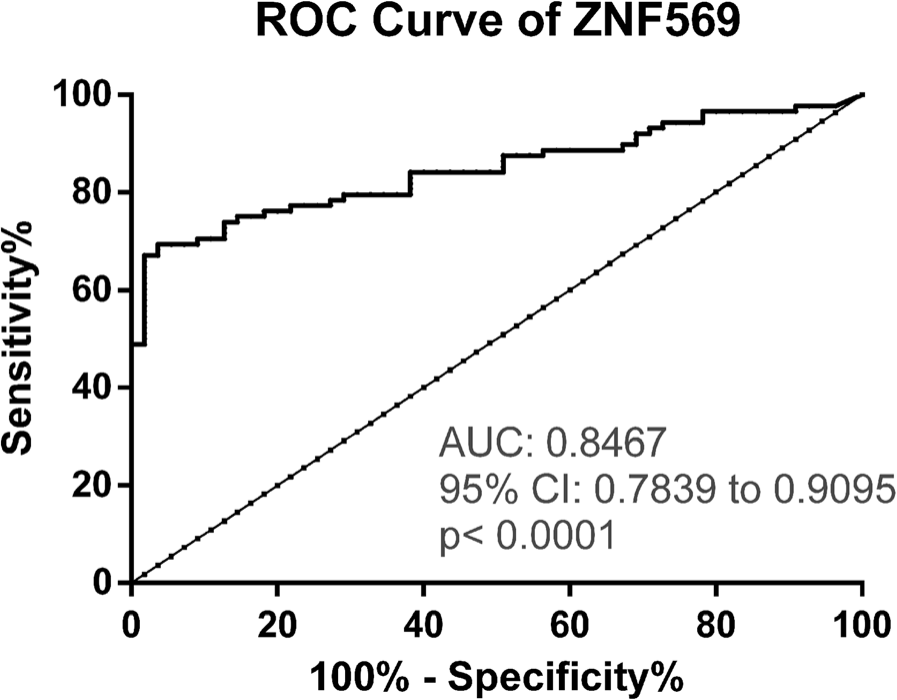
Receiver Operating Characteristic Curve of the *ZNF569* in Esophageal naïve tumors tissues

Using ROC curve analysis (Fig. 2), a cut-off of 55.17 was set for *ZNF569*me to assess biomarker performance. Thus, over 90% specificity and 69.3% sensitivity (Table 2) was disclosed, corresponding to an AUC of 0.8467 (Fig. 2).

**Table 2:** Performance of promoter gene methylation as biomarkers for detection of Esophageal Cancer

Gene	Sensitivity %	Specificity %	Accuracy %	LH +	LH-
*ZNF569*	69.3	96.4	79.7	19.06	0.32

### Association between promoter methylation levels and standard clinicopathologic features

**Fig. 3:**
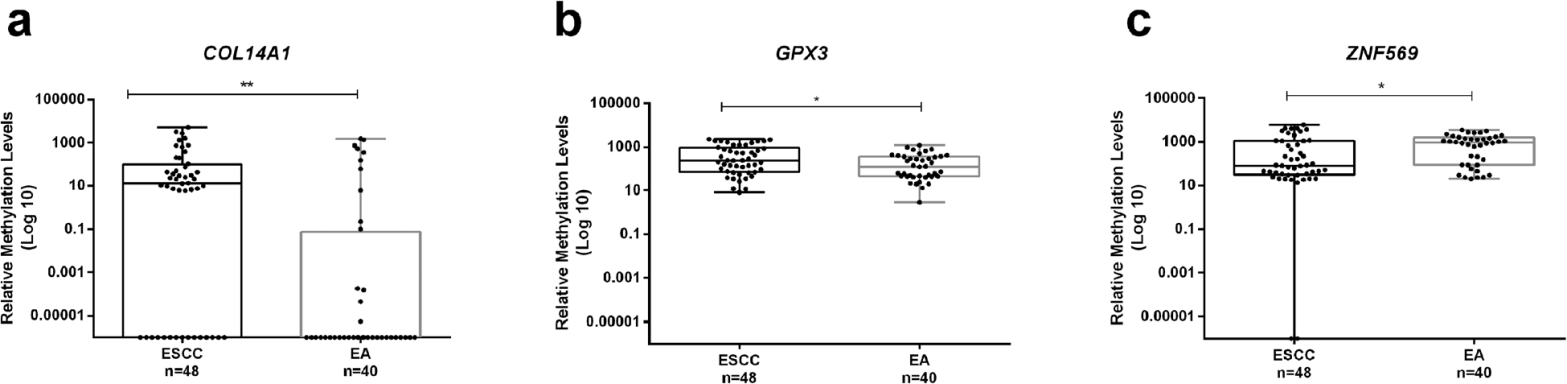
Boxplots with all points of *COL14A1* (**a**), *GPX3* (**b**) and *ZNF569* (**c**) relative methylation levels in Esophageal Squamous Cell Carcinoma (ESCC), *n* = 48 and Esophageal Adenocarcinoma (EA), *n* = 40 tissues. * *p* < 0.05; ** *p* < 0.01

*COL14A1*me, *GPX3*me, and *ZNF569*me levels were tested for associations with standard clinicopathological features in naïve ECa patients. All genes disclosed significant differences in promoter methylation levels according to histological subtype (Fig. 3). ESCC displayed higher *COL14A1*me and *GPX3*me levels than EA (*p* = 0.001 and *p* = 0.024, respectively), whereas EA displayed higher *ZNF569*me levels (*p* = 0.020). Additionally, *COL14A1*me levels were significantly higher in pT1 tumors compared to pT3 (*p* = 0.006) (Additional Fig. 1). However, no significant differences on methylation levels were found among different N stages (Additional Fig. 2).

### Biomarker performance according to histological subtype

**Fig. 4:**
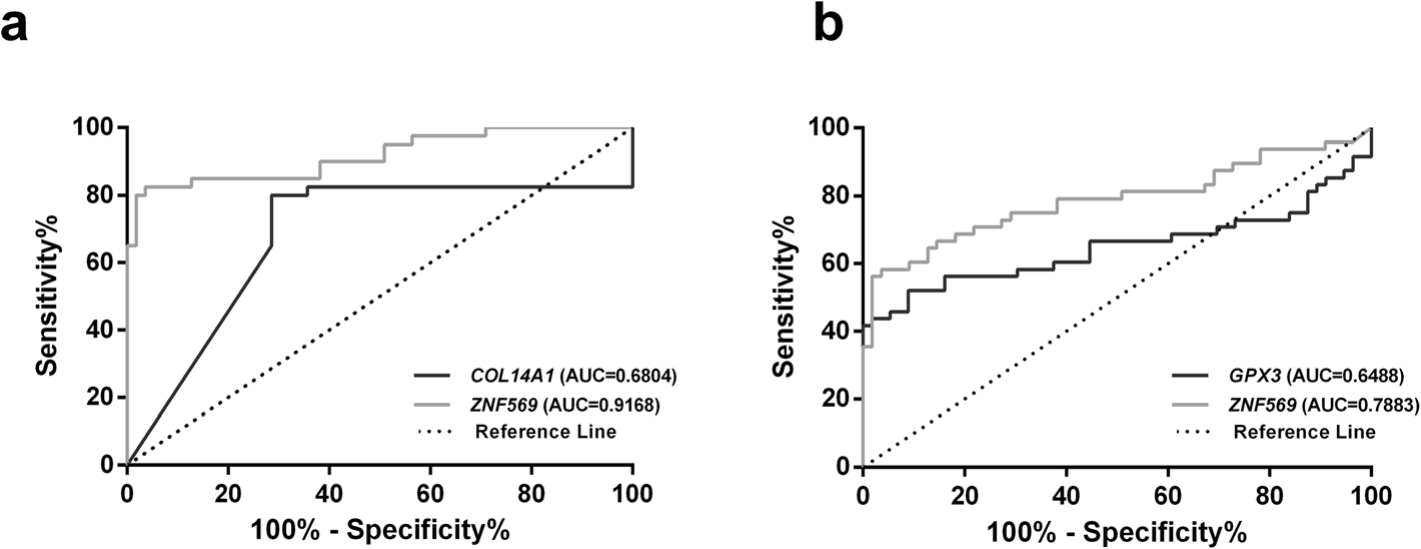
Receiver Operating Characteristic Curve of the two-gene panel (**a**) *COL14A1* and *ZNF569* in Esophageal Adenocarcinoma; (**b**) *GPX3* and *ZNF569* in Esophageal Squamous Cell Carcinoma

Because methylation levels differed between histological subtypes, samples were stratified according to this parameter for assessing histotype-specific biomarker performance. Concerning EA, *COL14A1*me and *ZNF569*me levels significantly differed from normal samples (*p =* 0.002 and *p* < 0.001, respectively). *COL14A1*me and *ZNF569*me levels identified EA with an AUC of 0.68 and 0.91, respectively (Fig. 4a), whereas the combination of both genes disclosed a sensitivity above 97% and 82.29 accuracy (Table 3). Furthermore, *GPX3*me and *ZNF569*me levels differed significantly between ESCC and normal samples (*p =* 0.009 and *p* < 0.001, respectively), individually discriminating this tumor type from controls with AUC of 0.65 and 0.79 (Fig. 4b), respectively. Accuracy of detection improved to 81.73 when the two genes were combined in a single panel (Table 3).

**Table 3:** Performance of promoter gene methylation as biomarkers for detection of Esophageal Cancer according histological subtype

	Gene	Sensitivity %	Specificity %	Accuracy %	LH +	LH-
EA^a^	*COL14A1*	80.00	71.43	75.00	2.80	0.28
	*ZNF569*	82.5	96.4	90.5	22.69	0.18
	Panel-EA	97.50	71.43	82.29	3.41	0.04
ESCC^b^	*GPX3*	52.1	91.132	73.1	5.83	0.53
	*ZNF569*	58.3	96.4	78.6	16.04	0.43
	Panel-ESCC	75.00	87.50	81.73	6.00	0.29

^a^*EA* Esophageal Adenocarcinoma, ^b^ *ESCC* Esophageal Squamous Cell Carcinoma

### Assessment of promoter methylation in post-treatment samples

**Fig. 5:**
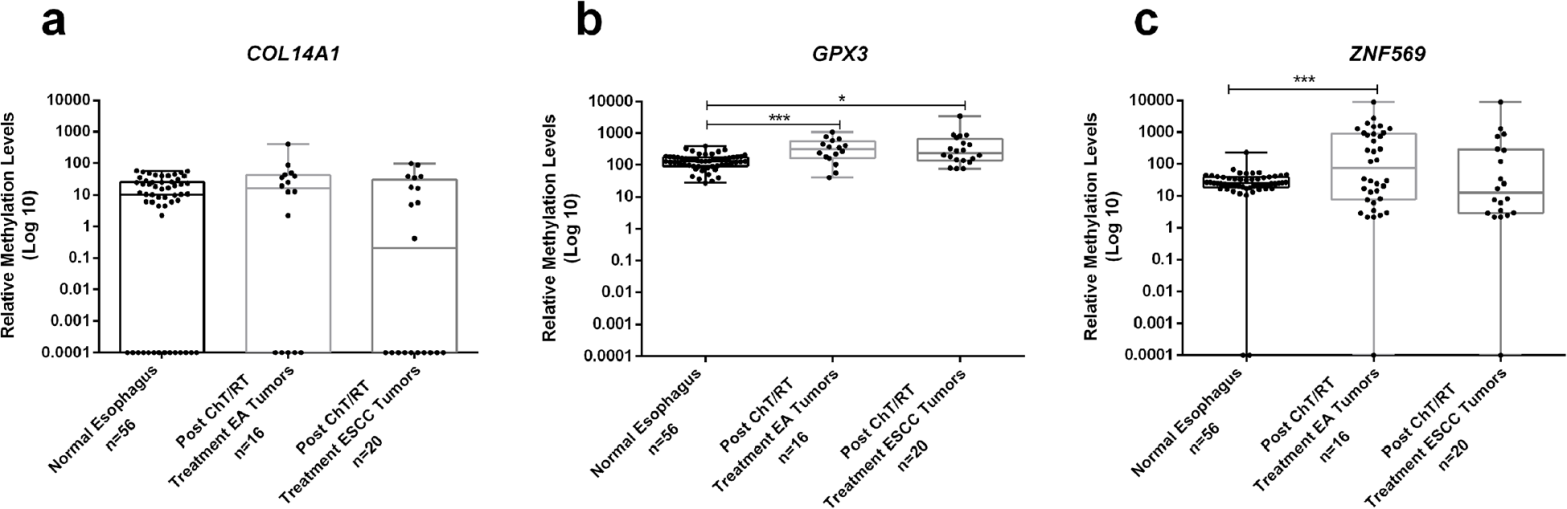
Boxplots with all points of *COL14A1* (**a**), *GPX3* (**b**) and *ZNF569* (**c**) relative methylation levels in the normal esophagus, *n* = 56, Esophageal Adenocarcinoma (EA), n = 16 and Esophageal Squamous Cell Carcinoma (ESCC), *n* = 20 Post-Chemotherapy and/or Radiotherapy patient’s samples. * *p* < 0.05; *** *p* < 0.001

*GPX3*me and *ZNF569*me levels were significantly higher in residual EA samples after neoadjuvant treatment compared to normal samples (*p* < 0.001, for both). Nonetheless, *COL14A1*me levels in tumors after neoadjuvant treatment did not differ significantly from normal samples (*p =* 0.493) (Fig. 5). Concerning ESCC, only *GPX3*me levels remained significantly different between post-treatment tumor and normal samples (*p =* 0.001).

### Survival analysis

Survival analysis was carried out in the treatment-naïve ECa cohort. For analysis, a maximum of 5 years follow-up was considered. During this period, 39 patients died from the disease (44.3%), three patients died with the disease (3.4%) and six patients died without evidence of cancer (6.8%). Among the remainder patients, 39 were alive without evidence of disease (44.3%) and one patient was alive with disease (1.2%).

No associations were depicted between *COL14A1*me*, GPX3*me, and *ZNF569*me levels and disease-specific or disease-free survival, whereas the pT stage, pN stage, and stage associated with both disease-specific and disease-free survival (*p* = 0.010, *p* = 0.002, *p* = 0.002 for disease-specific survival and *p* = 0.012, *p* = 0.001, *p* = 0.025 for disease-free survival).

## Discussion

ECa remains a leading cause of cancer-related mortality globally [1] and in Portugal [23]. Most patients are diagnosed with locally advanced or metastatic disease, entailing poor 5-years survival rate (about 25 and 5%, respectively) [24]. Thus, new strategies for early detection of this malignancy are urgently needed. In this context, epigenetic alterations such as DNA methylation have emerged as promising biomarkers in several cancers, including ECa [15, 25].

Herein, we tested three gene promoters’ methylation as ECa DNA methylation-based biomarkers, following a literature review. We selected the genes *ZNF569*, *GPX3,* and *COL14A1*, all previously reported to harbor promoter methylation and suggested to have an oncossuppressive function*.* ZNF569 protein has been reported as a potential transcriptional repressor implicated in MAPK signaling pathway [26]. *COL14A1* encodes for the alpha chain of type XIV collagen which interacts with decorin associated with cell growth and survival [27]. GPX3 is a glutathione peroxidase found to catalyze glutathione’s reduction of organic hydroperoxides and hydrogen peroxide and thereby protecting cells against oxidative damage [28, 29].

*COL14A1* aberrant methylation has been reported in ESCC [16], as well as in renal cell carcinoma, sarcomas, and endometrial carcinoma [27, 30, 31], whereas hypomethylation has been shown in coronary artery disease [32]. *GPX3* promoter methylation has been shown in ESCC and esophageal glandular lesions, including Barrett’s esophagus and EA [29, 33, 34]. In the same vein, *ZNF569* promoter hypermethylation has been associated with glandular lesions, like Barrett’s esophagus [17] in comparison with the normal esophagus.

Interestingly, we showed that *ZNF569me* levels could discriminate between ECa and normal esophagus with high specificity, regardless of the histotype, extending those previous reports. Furthermore, *ZNF569*me was shown to play a tumor-suppressive role in head and neck squamous cell carcinoma [35] and a DNA-methylation based panel, which included *ZNF569me*, discriminated gastric adenocarcinoma from normal mucosa [36].

ESCC and EA displayed different cancer-specific methylation patterns. Accordingly, differential methylation patterns have been previously reported between ESCC and EA [37, 38]. Indeed, in treatment-naïve tumors, the three selected genes disclosed different methylation levels among ESCC and EA, variably comparing to normal esophageal tissue samples. In particular, available data support the value of identification specific ESCC methylation panels to enable early detection [16, 39, 40]. We found *ZNF569* hypermethylated in both histological subtypes and, thus, this gene constitutes a promising biomarker for ECa detection, regardless of histological subtype. Moreover, we found *COL14A1* promoter methylation levels slightly higher in ESCC when compared with normal esophagus samples, although not statistically significant. This can be partially explained by variations in the population (Asian vs. Caucasian), as previously attested for some genes [41], and the different nature of samples tested (plasma vs. FFPE). Notwithstanding, *COL14A1* promoter methylation levels were significantly lower in EA compared to normal. To our knowledge, this is the first reported association between *COL14A1* methylation levels and EA. Conversely, *GPX3* promoter methylation levels did not differ between EA from normal tissues, although a few cases disclosed higher methylation levels (data not shown). Notwithstanding, different sample processing (fresh frozen tissues vs. FFPE) [29, 34], different methodologies to assess *GPX3* methylation levels among studies (qMSP vs. MSP vs. methylation ligation-dependent macroarray vs. pyrosequencing), and the smaller size of some cohorts [29, 33] may explain some disparate results.

Overall, we propose two different methylation-based panels, both with high accuracy to early detect ECa according to histological subtype. The ESCC-panel displayed higher specificity (87.5%), whereas the EA-panel disclosed higher sensitivity (97.5%). In fact, the performance of both methylation-based panels compares well with that of other studies [38, 42, 43]. For EA detection, Moinova et al. reported a two-gene methylation panel, comprising *CCNA1* and *VIM*, with higher specificity (91.7%), similar to *TAC1* hypermethylation reported by Jin et al. [44, 45]. Additionally, for ESCC Li et al. and Wang et al. reported panels with higher performance in Asian populations [16, 46].

Currently, most ECa patients are treated with neoadjuvant therapy followed by surgery, if diagnosed with locally advanced disease [2]. The randomized CROSS trial showed that surgery has a major impact on ECa patients’ QoL. Features such as fatigue and physical performance are decreased even in long-term survivors. Those effects are similar in patients undergoing neoadjuvant treatment or surgery only, emphasizing the impact of surgery in QoL [7, 8]. Hence, biomarkers enabling the identification of patients complete response to neoadjuvant treatment (who might be spared surgery) and to early detect disease recurrence are needed [47] to improve QoL without risking the likelihood of cure. Thus, we evaluated methylation levels of candidate genes in 36 samples from non-complete responders after neoadjuvant treatment. In our series, *ZNF569*me levels only significantly differed in EA comparatively to the normal esophagus, whereas *GPX3* promoter methylation levels were significantly higher both in ESCC and EA than in the normal esophagus. Because *GPX3me* has been associated with ChT resistance [28], *GPX3*me levels observed in EA after neoadjuvant treatment might be explained by selective pressure caused upon neoplastic cells, entailing adaptative alterations induced by treatment [48]. Several studies have associated DNA methylation with ChT or RT resistance [49–52]. However, most used samples before any treatment or in vitro studies with immortalized cell lines [51]. Thus, a direct comparison between our results and previously reported data should be made with caution. Nonetheless, the lack of information on methylation status before treatment, the small size of the pre-treated patient cohort along with the retrospective nature of our series, and the limited access to normal esophagus samples are major limitations of this study. Importantly, our series comprised ESCC and EA samples in similar proportions, contrarily to most of the previous studies which evaluated methylation status. Moreover, this study reported the potential of DNA methylation-based biomarkers for patients’ monitoring after neoadjuvant treatment.

## Conclusions

In conclusion, we identified two gene panels that might detect ESCC and EA with good accuracy, which might prove useful for early disease detection among high-risk populations, as well as to detect residual disease after neoadjuvant treatment. As future perspectives, we intend to validate these panels in liquid biopsies, using plasma samples as a minimally invasive approach, not only for ECa early detection and diagnosis but also to identify patients with residual disease after neoadjuvant treatment, which are the most likely to benefit from surgery.

## Supplementary Information

**Additional file 1: Additional Table 1** – List of The Genes Considered for This Study. **Additional Table 2** – Primers sequences and qMSP conditions for each gene studied in tissues samples. **Additional Fig. 1** – Boxplots with all points of *COL14A1* (a), *GPX3* (b) and *ZNF569* (c) relative methylation levels in the pT stage groups (pT1 *n* = 16, pT2 *n* = 14, pT3/pT4 *n* = 58). *** *p* < 0.001. **Additional Fig. 2** – Boxplots with all points of *COL14A1* (a), *GPX3* (b) and *ZNF569* (c) relative methylation levels in the pN stage groups (pN0 *n* = 39, pN1 *n* = 17 pN2 *n* = 21, pN3 *n* = 11).

## Data Availability

All data generated or analyzed during this study are included in this published article and its supplementary information files.

## Abbreviations

-LH: Negative likelihood ratio
+LH: Positive likelihood ratio
*ACTβ*: *β-actin*
AJCC: American Joint Committee on Cancer
AUC: Area Under the Curve
ChT: Chemotherapy
*COL14A1*: *Collagen Type XIV alpha 1 chain*
*COL14A1me*: *COL14A1* methylation
EA: Esophageal Adenocarcinomas
ECa: Esophageal cancer
ESCC: Esophageal Squamous Cell Carcinomas
FFPE: Formalin-fixed paraffin-embedded
*GPX3*: *Glutathione Peroxidase 3*
*GPX3me*: *GPX3* methylation
IPO-Porto: Portuguese Oncology Institute of Porto
QMSP: Quantitative methylation-specific PCR
QoL: Quality of Life
ROC: Receiver Operator Characteristic
RT: Radiotherapy
WHO: World Health Organization
*ZNF569*: *Zinc finger protein 569*
*ZNF569me*: *ZMF569* methylation
