# Supplementary material for: A DNA methylation-based test for esophageal cancer detection
Source: Biomark Res. 2020 Nov 25;8:68. doi: 10.1186/s40364-020-00248-7 (PMC7691099; doi:10.1186/s40364-020-00248-7)
Supplement: Supplementary file 1 — Additional file 1: Additional Table 1 – List of The Genes Considered for This Study. Additional Table 2 – Primers sequences and qMSP conditions for each gene studied in tissues samples. Additional Fig. 1 – Boxplots with all points of COL14A1 (a), GPX3 (b) and ZNF569 (c) relative methylation levels in the pT stage groups (pT1 n = 16, pT2 n = 14, pT3/pT4 n = 58). *** p < 0.001. Additional Fig. 2 – Boxplots with all points of COL14A1 (a), GPX3 (b) and ZNF569 (c) relative methylation levels in the pN stage groups (pN0 n = 39, pN1 n = 17 pN2 n = 21, pN3 n = 11). [file 40364_2020_248_MOESM1_ESM.docx]

Salta et al-ADDITIONAL File- 1

**Additional Table 1 – List of The Genes Considered for This Study.**

**Additional Table 2 – Primers sequences and qMSP conditions for each gene studied in tissues samples.**

**Additional Figure Legends:**

**Additional Figure 1 – Boxplots with all points of *COL14A1* (a), *GPX3* (b) and *ZNF569* (c) relative methylation levels in the pT stage groups (pT1 n=16, pT2 n=14, pT3/pT4 n=58). *** p<0.001.**

**Additional Figure 2 – Boxplots with all points of *COL14A1* (a), *GPX3* (b) and *ZNF569* (c) relative methylation levels in the pN stage groups (pN0 n=39, pN1 n=17 pN2 n=21, pN3 n=11).**

Additonal Tables

Additional Table 1 – List of the genes considered for this study.

| Gene | Sensitivity (%) | Specificity (%) | AUC^a^ (%) | Specimen type | Methods | Cases  (n) | Controls (n) | Exclusion criteria | Ref |
| --- | --- | --- | --- | --- | --- | --- | --- | --- | --- |
| *TFPI2* | 78.52 | 96.9 | 87.7 | Biopsies and cytosponge samples | MethyLight PCR | 149 BE^b^ | 129 | Lower Specificity | (1) |
| *TWIST1* | 69.8 | 93.02 | 81.4 |  |  |  |  | Lower Specificity |  |
| *ZNF345* | 62.42 | 100 | 81.2 |  |  |  |  | Technical Limitations |  |
| *ZNF569* | 59.06 | 99.22 | 78.7 |  |  |  |  | --- |  |
| *EPB41L3* | 31 | 100 | 65.5 | Tissues and Blood samples | MSP^c^/ Methylation 450k array | 42 ESCC^d^ | 50 | Technical Limitations | (2) |
| *GPX3* | 40.5 |  | 70.2 |  |  |  |  | --- |  |
| *COL14A1* | 31 |  | 65.5 |  |  |  |  | --- |  |
| *STK3* | 66 | 77 | 71 | Peripheral blood mononuclear cells and peripheral blood leukocytes | Bisulfite Sequencing Method | 94 ESCC | 94 | Lower Specificity | (3) |
| *ZNF418* | 58 | 93 | 79 |  |  |  |  |  |  |
| *ZNF542* | 72 | 82 | 83 |  |  |  |  |  |  |
| *PAX1* | 100 | 78.6 | 89.3 | FFPE | MethyLight PCR | 14 ESCC | 14 | Small cohort size | (4) |
| *ZNF582* | 85.7 | 100 | 95.4 |  |  |  |  |  |  |
| *PAX1* | 80.7 | 75.0 | 76.0 | TCGA | MethyLight PCR | 187 | 16 | Small cohort size |  |
| *ZNF582* | 88.2 | 81.2 | 80.7 |  |  |  |  |  |  |
| B3GAT2 | 50.0 | 100 | 94.5 | Endoscopic brushing | MethyLight PCR/ Methylation 450k array | 10 BE | 30 | Small cohort size | (5) |
| ZNF793 | 70.0 | 100 | 95.9 |  |  |  |  |  |  |
| *CCNA1* | 72 | 100 | 91.7 | Esophageal balloon sampling (cytological samples) | Bisulfite Sequencing Method | 50 cases (31 NDBE^e^, 11 BE, 8 EA^f^) | 36 | Small cohort size | (6) |
| *VIM* | 80 | 91.7 | 90.8 |  |  |  |  |  |  |
| *CASZ1* | 100 | 100 | 100 | Plasma Samples | Methylation 450k array | 10 ESCC | 10 | Small cohort size | (7) |
| *CDH13* | 100 | 100 | 100 |  |  |  |  |  |  |
| *ING2* | 100 | 100 | 100 |  |  |  |  |  |  |

Selected genes highlighted in light blue. **Abbreviations:** ^a^ AUC – Area Under de Curve; ^b^ BE – Barrett’s Esophagus; ^c^ MSP – Methylation Specific-PCR; ^d^ ESCC – Esophageal Squamous Cells Carcinoma; ^e^ NDBE – Non dysplastic Barrett’s esophagus; ^f^ EA – Esophageal Adenocarcinoma

1. Chettouh H, Mowforth O, Galeano-Dalmau N, Bezawada N, Ross-Innes C, MacRae S, et al. Methylation panel is a diagnostic biomarker for Barrett's oesophagus in endoscopic biopsies and non-endoscopic cytology specimens. Gut. 2018;67(11):1942-9.

2. Li X, Zhou F, Jiang C, Wang Y, Lu Y, Yang F, et al. Identification of a DNA methylome profile of esophageal squamous cell carcinoma and potential plasma epigenetic biomarkers for early diagnosis. PloS one. 2014;9(7):e103162.

3. Pu W, Wang C, Chen S, Zhao D, Zhou Y, Ma Y, et al. Targeted bisulfite sequencing identified a panel of DNA methylation-based biomarkers for esophageal squamous cell carcinoma (ESCC). Clinical epigenetics. 2017;9:129.

4. Huang J, Wang G, Tang J, Zhuang W, Wang LP, Liou YL, et al. DNA Methylation Status of PAX1 and ZNF582 in Esophageal Squamous Cell Carcinoma. Int J Environ Res Public Health. 2017;14(2).

5. Yu M, O'Leary RM, Kaz AM, Morris SM, Carter KT, Chak A, et al. Methylated B3GAT2 and ZNF793 Are Potential Detection Biomarkers for Barrett's Esophagus. Cancer epidemiology, biomarkers & prevention : a publication of the American Association for Cancer Research, cosponsored by the American Society of Preventive Oncology. 2015;24(12):1890-7.

6. Moinova HR, LaFramboise T, Lutterbaugh JD, Chandar AK, Dumot J, Faulx A, et al. Identifying DNA methylation biomarkers for non-endoscopic detection of Barrett's esophagus. Science translational medicine. 2018;10(424):eaao5848.

7. Wang HQ, Yang CY, Wang SY, Wang T, Han JL, Wei K, et al. Cell-free plasma hypermethylated CASZ1, CDH13 and ING2 are promising biomarkers of esophageal cancer. J Biomed Res. 2018;32(5):424-33.

**Additional Table 2 – Primers sequences and qMSP conditions for each gene studied in tissues samples**

| **Gene** | **Primers** | **Final concentration** | **Ref** |
| --- | --- | --- | --- |
| ***ACTβ*** | **F – 5’ TGG TGA TGG AGG AGG TTT AGT AAG T 3’** | **200nM** | **(1)** |
|  | **R – 5’ ACC AAT AAA ACC TAC TCC TCC CTT AA 3’** |  |  |
| ***COL14A1*** | **F – 5’ GTG AAT GGG TGT TTT TTT AGA TTT C 3’** | **250nM** | **(2)** |
|  | **R – 5’ AAC GCC TTT CGA CTT CTA CG 3’** |  |  |
| ***GPX3*** | **F – 5’ CGT TCG TTT TTG AAA TTT TAG TC 3’** | **250nM** | **(2)** |
|  | **R – 5’ CTA CCT AAT CCC TAA CCA CCG T 3’** |  |  |
| ***ZNF569*** | **F – 5’ GAA GTT AGT TTC GTT CGG GTG AGT 3’** | **250nM** | **(3)** |
|  | **R – 5’ GAC ACT AAA ACC GAC GCT ATC GAT 3’** |  |  |

1. Salta S, S PN, Fontes-Sousa M, Lopes P, Freitas M, Caldas M, et al. A DNA Methylation-Based Test for Breast Cancer Detection in Circulating Cell-Free DNA. Journal of clinical medicine. 2018;7(11).

2. Li X, Zhou F, Jiang C, Wang Y, Lu Y, Yang F, et al. Identification of a DNA methylome profile of esophageal squamous cell carcinoma and potential plasma epigenetic biomarkers for early diagnosis. PloS one. 2014;9(7):e103162.

3. Chettouh H, Mowforth O, Galeano-Dalmau N, Bezawada N, Ross-Innes C, MacRae S, et al. Methylation panel is a diagnostic biomarker for Barrett's oesophagus in endoscopic biopsies and non-endoscopic cytology specimens. Gut. 2018;67(11):1942-9.

**Additional Figure 1**

**

**

**Additional Figure 2**

**

**
